# A Book Reduction

**Published:** 1895-09

**Authors:** 


### A Book Reduction.

A notice has just come to hand from P. Blakiston Son, & Co., in which it is stated that they will in the future make the prices of all their medical, and of course dental books as well, at absolutely net prices. So that physicians and dentist will be able to purchase their books at the same price all over the United States. This will certainly be an inducement to purchase books to a greater extent than heretofore. Doubtless others in the book trade will make the same changes.

A reduction of twenty to thirty per cent. means much to the physician or dentist. *Make good selections, and buy books.*

